# Adverse Effects of Andrographolide Derivative Medications Compared to the Safe use of Herbal Preparations of *Andrographis paniculata*: Results of a Systematic Review and Meta-Analysis of Clinical Studies

**DOI:** 10.3389/fphar.2022.773282

**Published:** 2022-01-28

**Authors:** Ya-xi Shang, Chen Shen, Trine Stub, Si-jia Zhu, Shu-yu Qiao, Yu-qi Li, Rui-ting Wang, Jing Li, Jian-ping Liu

**Affiliations:** ^1^ Centre for Evidence-Based Chinese Medicine, Beijing University of Chinese Medicine, Beijing, China; ^2^ The National Research Center in Complementary and Alternative Medicine (NAFKAM), Department of Community Medicine, Faculty of Health Science, UiT, The Arctic University of Norway, Tromsø, Norway; ^3^ Dongzhimen Hospital, Beijing University of Chinese Medicine, Beijing, China; ^4^ Beijing Key Laboratory of the Innovative Development of Functional Staple and the Nutritional Intervention for Chronic Disease, China National Research Institute of Food and Fermentation Industries Co, Ltd., Beijing, China

**Keywords:** *Andrographis paniculata*, andrographolide, drug safety, adverse drug reaction, adverse event, clinical study

## Abstract

**Background and objective:** *Andrographis paniculata* (AP) is a traditionally used herbaceous plant, whose main active constituent is andrographolide. Andrographolide derivative medications and herbal preparations of AP are often used to treat respiratory tract infections. This study aims to systematically evaluate the safety of andrographolide derivative medications and herbal preparations of AP based on clinical studies.

**Methods:** English and Chinese databases were searched for all types of clinical studies that reported adverse drug reactions (ADRs) and adverse events (AEs) of andrographolide derivative medications and herbal preparations of AP. The ADRs and AEs were classified according to manifestations, and graded according to severity. Single-rate meta-analysis was performed for ADR incidence using R software.

**Results:** A total of 262 studies were included, including 125 randomized controlled trials, 23 non-randomized controlled trials, 6 case series, and 108 case reports. In 9490 participants using andrographolide derivative injections, 383 (4.04%) reported ADRs. Meta-analysis showed that the ADR incidence of three most frequently used injections of andrographolide derivatives (andrographolide sulfonate, potassium sodium dehydroandrographolide succinate, and potassium dehydroandrographolide succinate) were 5.48% [95% CI (4.47%, 6.72%)], 3.69% [95% CI (2.59%, 4.94%)] and 5.33% [95% CI (3.68%, 7.72%)], respectively, which may be slightly higher than the actual ADR incidence, because only studies that reported the occurrence of ADRs or AEs were included, but studies without ADR and AE were not included. The ADRs of andrographolide derivative injections were mainly gastrointestinal, skin and subcutaneous tissue disorders, and anaphylaxis. Fifty-five patients experienced life-threatening anaphylactic shock, three patients died, and the causation attributed to the andrographolide derivative injection. Other ADRs were mild, moderate or medically significant. Nine herbal preparations of AP were tested in 10 studies, and the reported ADRs were mainly mild to moderate gastrointestinal, skin and subcutaneous tissue disorders. Except for five patients using andrographolide derivative injections eventually died, most of the ADRs were alleviated after drug withdrawal and symptomatic treatment.

**Conclusions:** The ADRs of andrographolide derivative medications are few, but can be life-threatening, mainly gastrointestinal, skin and subcutaneous tissue disorders, and anaphylaxis. Injections of andrographolide derivatives should be used with caution. Herbal preparations of AP are essentially safe.

**Systematic Review Registration**: [website], identifier [registration number]

## Introduction

*Andrographis paniculata* (Burm.f.) Nees (AP) is an herbaceous plant in the Acanthaceae family, and has been traditionally used in China and India (Akbar, 2011). AP has the pharmacological effects of anti-inflammatory, antibacterial, antiviral, antihyperglycemic, anticancer, antistress, hepatoprotective, and immunomodulatory (Hossain et al., 2014; Panossian et al., 2021). Herbal preparations of AP have been used in many countries to treat respiratory tract infections (RTIs) (Thamlikitkul et al., 1991; Cáceres et al., 1999; Saxena et al., 2010) and colitis (Tang et al., 2011; Sandborn et al., 2013), to treat early stages of COVID-19 (Kuchta et al., 2021), and to relieve the symptoms of arthritis (Burgos et al., 2009; Hancke et al., 2019). Results of previous systematic reviews showed that, herbal preparations of AP were beneficial and safe for relieving the symptoms of RTIs and shortening time to symptom resolution (Coon and Ernst, 2004; Hu et al., 2017). Andrographolide, a labdane diterpenoid, is the main active constituent of AP (Chakravarti and Chakravarti, 1951). Andrographolide has the pharmacological effect of anti-inflammatory (Tan et al., 2017), anti-viral (Paemanee et al., 2019), anti-bacterial (Zhang et al., 2020), anticancer (Khan et al., 2018), and hepatoprotective (Singha et al., 2007). Andrographolide is insoluble in water and non-polar solvents (Sareer et al., 2014). The low water solubility limits its therapeutic use. Some chemical derivatives of andrographolide are soluble in water, and can be more widely used in clinical practice. In China, injections of andrographolide derivatives on the market have been used for the treatment of various diseases, such as upper respiratory tract infections (URTIs), pneumonia, hand, foot and mouth disease, and COVID-19 (Zhang et al., 2017; Li et al., 2019; Shi et al., 2019; Sun et al., 2019; Zhang et al., 2021).

Adverse event (AE) is an unfavourable outcome that occurs during or after the use of a drug or other intervention but is not necessarily caused by it. Adverse drug reaction (ADR) is an adverse event for which the causal relationship between the drug and the event is at least a reasonable possibility (Higgins and Green, 2011). According to previously published studies, the ADRs of andrographolide derivative injections include those of skin and mucous membranes (rash, itching, edema, flushing), digestive system (nausea, vomiting, abdominal pain, diarrhea), blood system (thrombocytopenia, leukopenia), circulatory system (chest tightness, palpitations), respiratory system (dyspnea, anhelation, cough), and nervous system (dizziness, headache, convulsion, coma) (Xiang et al., 2016). Previous studies have shown that the ADRs of AP herbal preparations include nausea, vomiting, diarrhea, abdominal pain, dizziness, epistaxis, rash (Saxena et al., 2010; Li and Yang, 2016).

In the published systematic reviews of andrographolide derivative injections, some reviews reported about safety, but they only briefly stated the ADRs and AEs, and did not systematically review the information of these ADRs and AEs. A few studies systematically reviewed the safety of single andrographolide derivative (andrographolide sulfonate, potassium sodium dehydroandrographolide succinate, and andrographolide sodium bisulfite) (Yang et al., 2013; Chen et al., 2016; Gao et al., 2017). One study reviewed the safety of four injections of andrographolide derivatives (Xiang et al., 2016). No systematic review has evaluated the safety of AP herbal preparations. Therefore, in order to comprehensively review and evaluate the safety of andrographolide derivative medications and herbal preparations of AP, we conducted this systematic review.

## Methods

### Search Strategy

We conducted systematic searches in seven electronic databases, including four Chinese databases (China National Knowledge Infrastructure (CNKI), Wanfang Database, Chinese Scientific Journal Database (VIP) and SinoMed) and three English databases (PubMed, EMBASE and the Cochrane Library), from their inception to January 2021. Search terms such as “*Andrographis* (Chuanxinlian)”, “andrographolide”, “adverse drug reaction”, “adverse event” and “safety” were used. The reference lists of studies meeting the inclusion criteria were also searched to identify additional relevant studies. A detailed search strategy for PubMed is attached as Supplementary Material.

### Study Selection

Four authors in pair (Y-xS, S-jZ, S-yQ, CS) screened the literature independently. Any disagreement was resolved by discussion with a third author (J-pL). Clinical studies that meet all the following criteria will be included: 1) Clinical studies using andrographolide derivative medications or herbal preparations of AP; 2) Clinical studies that reported the occurrence of ADRs or AEs after using these preparations. 3) There are no restrictions on the characteristics of participants, control groups and study types. Studies that meet the following criteria will be excluded: repeated studies, or studies that used other preparations in addition to andrographolide derivative medications, herbal preparations of AP, and symptomatic treatment.

### Data Extraction and Quality Assessment

Six authors in pair (Y-xS, S-jZ, S-yQ, CS) extracted data and assessed the methodological quality of the included studies independently. Any disagreement was resolved by discussion. The extracted information mainly includes: study type, demographic characteristics of participants, details of interventions (preparation name, dosage, course of treatment) and the information of ADRs and AEs.

For different types of studies, corresponding tools were used to evaluate the methodological quality. For randomized controlled trials (RCTs), the risk of bias tool was used (Higgins and Green, 2011). Items including random sequence generation, allocation concealment, blinding of participants and personnel, blinding of outcome assessment, incomplete outcome data and selective reporting were judged as “low risk”, “high risk” or “unclear risk”. For non-randomized controlled trials (non-RCTs), the MINORS (Methodological Index for Non-Randomized Studies) tool was used (Slim et al., 2003). Twelve items were rated as 0 point (not reported), 1 point (reported but inadequate) or 2 points (reported and adequate) respectively. Studies with a score of 18 or higher were rated as high-quality studies. For cohort studies, the Newcastle-Ottawa Scale was used (Wells et al., 2021), which consists of eight items related to selection, comparison and outcome. For case series, quality assessment tool recommended by the National Institute for Clinical Excellence (NICE) was used (Yu et al., 2008). Studies reporting more than six of the eight items were rated as high-quality studies.

We evaluated the causal relationship between andrographolide derivative medications/herbal preparations of AP and the adverse events, according to the World Health Organization-Uppsala Monitoring Centre (WHO-UMC) causality assessment criteria (Centre, 2013). Adverse events that were certainly, probably, and possibly related to andrographolide derivative medications/herbal preparations of AP were judged as ADRs.

The Common Terminology Criteria for Adverse Events (CTCAE) system was used to classify the ADRs and AEs according to manifestations, and grade them according to severity (Services, 2017). The CTCAE system grades adverse events from 1 to 5, where 1 is mild, 2 is moderate, 3 is severe or medically significant, 4 is life-threatening, and 5 is lethal. The severity of ADRs and AEs were graded based on the information provided in the articles. When reporting the severity grading of ADRs and AEs, we reported the number of ADRs and AEs, not the number of patients experiencing the ADRs and AEs.

### Data Analysis

The R3.6.1 software was used for data analysis. Single-rate meta-analysis was performed for the ADR incidence of included studies, if the studies were similar in intervention. Results were considered homogenous when the I^2^ statistic was less than 50%, and the p-value for the test of heterogeneity was ≥0.10. In these cases, a fixed-effect model was used to compute the pooled estimate of ADR incidence. In all other cases, the studies were considered heterogeneous, and a random-effect model was used to compute the pooled ADR incidence. Other data not suitable for pooling analysis were synthesized qualitatively.

## Results

### Description of Included Studies

**FIGURE 1:**
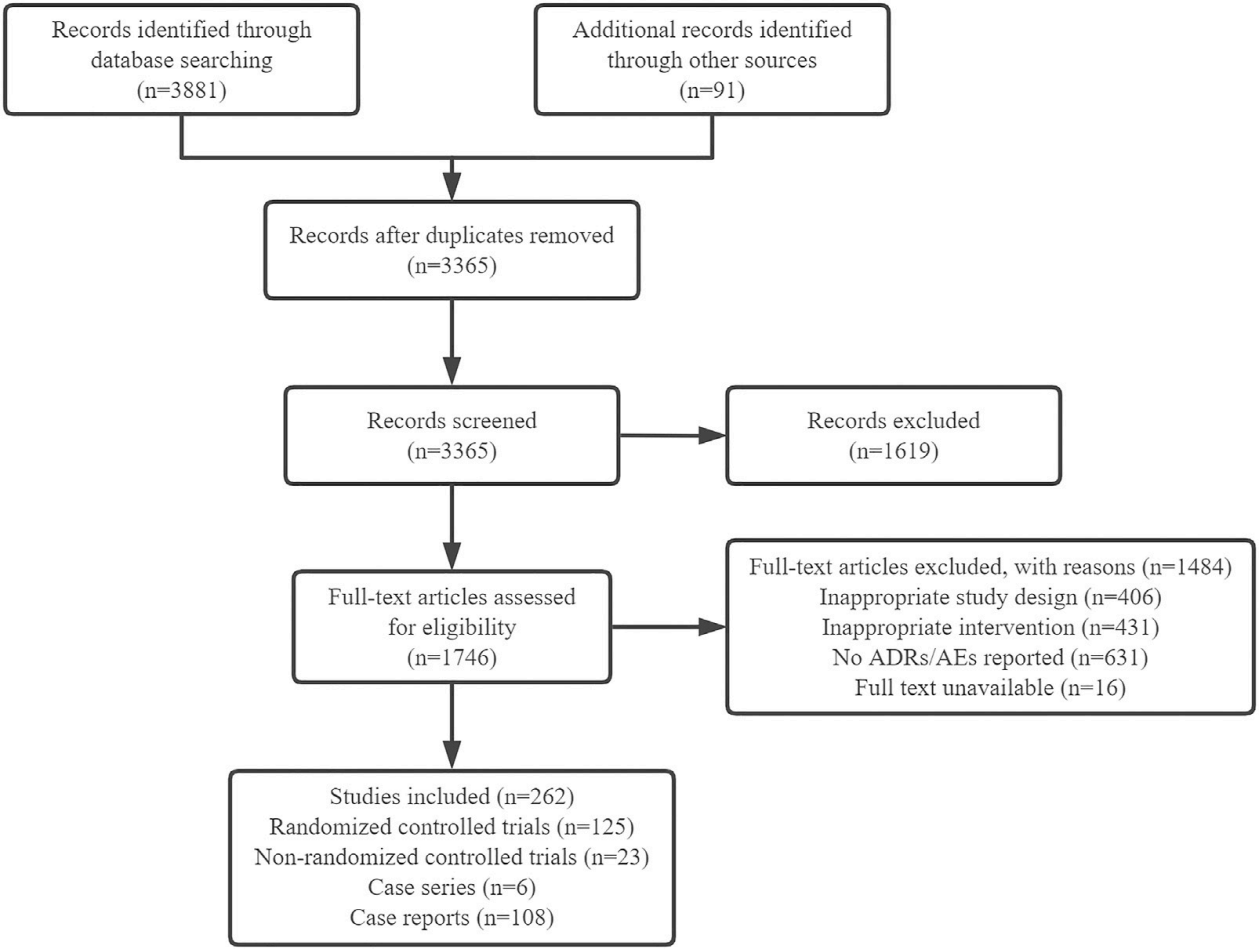
Flow diagram of study selection.

The literature search identified 3972 citations, of which 607 were excluded due to duplication. After reviewing the titles and abstracts, 1619 citations were excluded. After scanning the full text, 1484 publications were excluded. Finally, we included 262 studies, including 125 RCTs, 23 non-RCTs, six case series and 108 case reports (Figure 1). Most of the studies were published in Chinese, only nine RCTs and three case series were published in English.

The sample size of included studies ranged from 20 to 2000, except for case reports. The age of participants ranged from 1 month to 88 years old. The included RCTs, non-RCTs and case series focused on URTIs (43.51%), pneumonia (15.58%), intestinal infections (12.34%), hand-foot-mouth disease (6.50%), bronchitis (5.84%), other RTIs (5.84%) and other diseases (10.39%) such as encephalitis, herpes, multiple sclerosis, hypertriglyceridemia, chronic obstructive pulmonary diseases, knee osteoarthritis, rheumatoid arthritis, type 2 diabetes mellitus and unspecified bacterial infections. The included case reports involved a total of 155 cases, of which 58.71% were URTIs, 14.84% were bronchitis, 5.81% were pneumonia, 7.74% were other RTIs, 2.58% were intestinal infections, 9.03% did not report specific diagnosis, and other cases were lung cancer and arthritis.

### Quality of Included Studies

**FIGURE 2:**
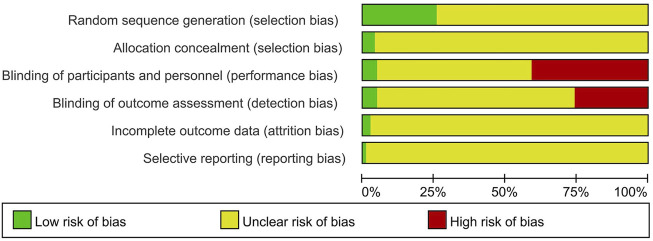
Risk of bias of RCTs graph.

Among the included RCTs, 32 studies were assessed with low risk of bias in random sequence generation, of which 27 used random number table, three used computer software (Burgos et al., 2009; Hancke et al., 2019; Ciampi et al., 2020), one used the method of tossing coin (Xian et al., 2016), and one used the method of lottery (Phunikhom et al., 2015), to generate random sequence. Other RCTs (*n* = 93) did not report the method of random sequence generation. Among the 125 RCTs, only five trials provided information on allocation concealment (Burgos et al., 2009; Saxena et al., 2010; Sandborn et al., 2013; Phunikhom et al., 2015; Hancke et al., 2019). Of them, two used central allocation (Saxena et al., 2010; Sandborn et al., 2013), and three used opaque and sealed envelopes (Burgos et al., 2009; Phunikhom et al., 2015; Hancke et al., 2019). Other studies (*n* = 120) did not provide the information of allocation concealment. Among the included RCTs, 51 were assessed with high risk of bias due to insufficient blinding procedures of participants and personnel, because the interventions between treatment group and control group were different in preparation, dosage or frequency. Six trials were placebo-controlled (Burgos et al., 2009; Saxena et al., 2010; Sandborn et al., 2013; Bertoglio et al., 2016; Hancke et al., 2019; Ciampi et al., 2020), and were assessed with low risk of bias in blinding of participants and personnel. The remaining trials (*n* = 68) did not provide enough information on blinding of participants and personnel. The majority (*n* = 87) of the included RCTs did not provide enough information on blinding of outcome assessment. Thirty-two trials were assessed to be at high risk of bias as they assessed subjective outcome measures and the participants or practitioners were not blinded. Six trials assessed subjective outcome measures, and the patients and practitioners did not know which study group they had been assigned to (Burgos et al., 2009; Saxena et al., 2010; Sandborn et al., 2013; Bertoglio et al., 2016; Hancke et al., 2019; Ciampi et al., 2020), and therefore were assessed with low risk of bias in blinding of outcome assessment. Three trials reported dropouts, and the dropout rate in two groups were approximate (Saxena et al., 2010; Phunikhom et al., 2015; Hancke et al., 2019). Other trials (*n* = 122) did not provide information on incomplete outcome data. One trial had a protocol that could be obtained, and the outcome measures were consistent with the trial report (Bertoglio et al., 2016). Other trials (*n* = 124) did not provide enough information on selective reporting (Figure 2).

All included non-RCTs (*n* = 23) provided the information on clearly stated aim, appropriate endpoints, follow up, contemporary groups and equivalent baseline. Among these included trials, 20 reported follow-up period, 19 reported information on statistical analyses, 15 reported adequate control group, and only two reported the information on prospective data collection (Zou, 2008; Tong, 2015). All these included trials did not provide information on inclusion of consecutive patients, unbiased assessment of endpoint, and prospective calculation of sample size. All trials scored between 11 and 14 points, and had a low overall methodological quality.

All the included case series (*n* = 6) clearly described the aim of the study, but did not report the information on prospective data collection and consecutive recruitment of patients. Among these case series, one was multicentered (Calabrese et al., 2000), four clearly reported the inclusion and exclusion criteria (Calabrese et al., 2000; Agarwal et al., 2005; Wu, 2008; Suriyo et al., 2017), two provided clear definition of outcomes (Calabrese et al., 2000; Suriyo et al., 2017), three clearly described the main findings of the study (Calabrese et al., 2000; Wu, 2008; Li and Yang, 2016), and two stratified the outcomes (Calabrese et al., 2000; Li and Tan, 2015). Only one study reported six items, and was of high quality (Calabrese et al., 2000). Other studies (*n* = 5) reported two to four items, and were of lower quality. The most commonly reported items were aim of the study and main findings of the study.

### Safety of Andrographolide Derivative Medications

Among the included RCTs, non-RCTs, and case series, a total of 142 studies (117 RCTs, 23 non-RCTs, and two case series) investigated injections of andrographolide derivatives, of which 75 (52.82%) investigated andrographolide sulfonate (AS, trade name: Xiyanping injection), 54 (38.03%) investigated potassium sodium dehydroandrographolide succinate (PSDS, trade name: Yanhuning injection), and nine (6.34%) used potassium dehydroandrographolide succinate (PDS, trade name: Chuanhuning injection). One study (0.70%) researched andrographolide sodium bisulfite (ASB, trade name: Lianbizhi injection) (Bi, 2006), and one (0.70%) investigated injection of Andrographis paniculata (trade name: Chuanxinlian injection) (Ma et al., 2009). The remaining two studies (1.41%) researched other injections (Liang, 1998; Yu et al., 2010). In these two studies, the authors only reported that the injections they used contained andrographolide, but did not specifically report the name and active ingredients of the injections.

For AS, the dosage stated in drug instruction is: 250–500 mg/day for adults and 5–10 mg/(kg.d) for children when administered by intravenous (IV) injection; 50–100 mg/time, 2–3 times/day for intramuscular (IM) injection in adults. For PSDS, the dosage stated in instruction is 0.16–0.4 g/day, IV, for adults. And for PDS, the dosage is 400–800 mg/day, IV, and 100 mg/time, IM, for adults. Among the 75 studies investigating AS, 67 (89.33%) used the injection in accordance with the dosage stated in the instruction. Three studies (4.00%) administered the injection at a dosage lower than the stated dosage (Su and Ke, 2012; Zhang, 2018; Zhu et al., 2018). Of these three studies, one was for patients after thoracotomy (dose: 100 mg/day, IV) (Zhu et al., 2018), one was for elderly patients (dose: 50 mg/day, IV) (Zhang, 2018), and one was for children (dose: 2.5–5 mg/(kg.d), IV) (Su and Ke, 2012). In two studies (2.67%) (Tong, 2015; Huang, 2016), the injection was used at an off-label dose, with doses of 7.5–12.5 mg/(kg.d) and 10–15 mg/(kg.d), IV, for children. In the remaining three studies (4.00%), whether the injection was used according to the instruction was unclear. Of these three studies, two studies included adults and children, but did not report the dosages for adults and children separately; and one study used the injection for aerosol inhalation treatment (Liu and Tian, 2012). Among the 54 studies using PSDS, six studies (11.11%) used the injection in accordance with the dosage prescribed in the instruction. One study (1.85%) used the injection at an off-label dose (400–600 mg/day, IV, for children) (Meng, 2005). In the remaining 47 studies (87.04%) for children, or for both adults and children, whether the injection was used in accordance with the instruction is unclear, because there is no specific prescribed dosage for children in the instruction of PSDS. Among the nine studies investigating PDS, five studies used it in accordance with the dosage stated in the instruction. In the other four studies which included children, it is unclear whether the injection was used according to the instruction, because there is no specific dosage for children in the instruction of PDS. In the two studies using ASB and injection of *Andrographis paniculata* for children (Bi, 2006; Ma et al., 2009), it is also unclear whether the injections were used according to the instructions, because there is no specific dosage for children in the instructions of these two injections. In these 142 studies, the treatment duration ranged from 2 days to 21 days, of which 119 studies (83.80%) had the treatment duration of 2–7 days, 10 studies (7.04%) had the duration of 8–14 days, one study had a treatment duration of 21 days (Wu, 2013), and the remaining 12 studies did not report the treatment duration. In 92 studies, the injections were used in combination with other symptomatic treatments (such as oxygen inhalation, physical cooling therapy, antipyretic therapy, rehydration therapy, phlegm-resolving and cough-relief treatment, and gastrointestinal mucosal protection therapy). In the other 50 studies, the injection was not used in combination with any other therapies.

**FIGURE 3:**
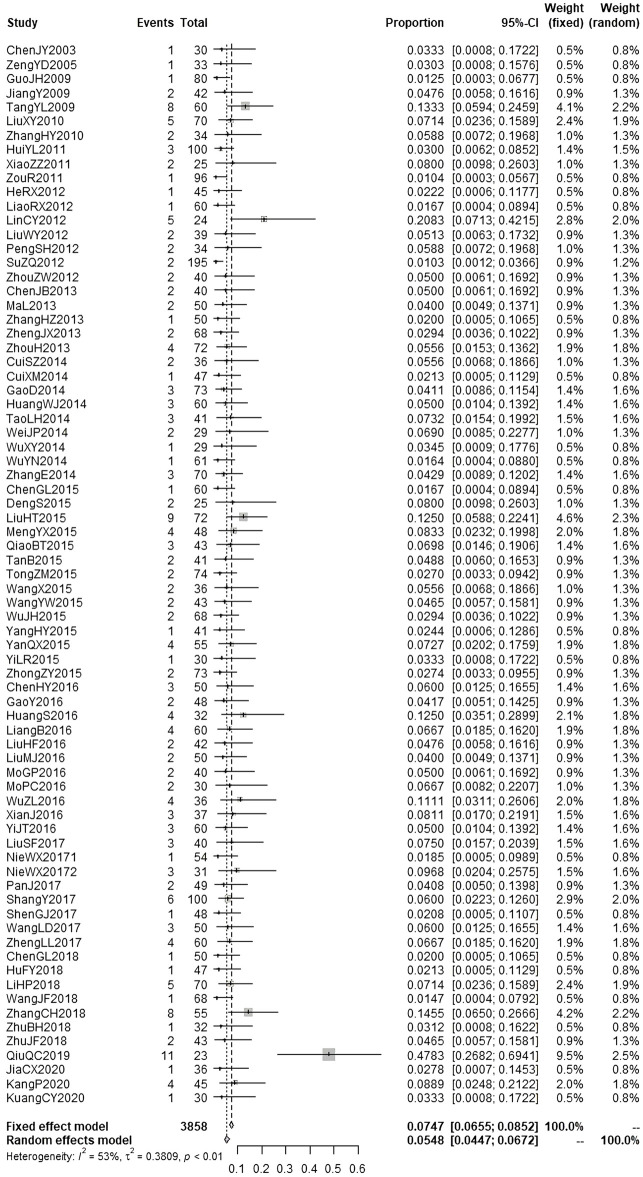
Meta-analysis on ADR incidence of AS.

**FIGURE 4:**
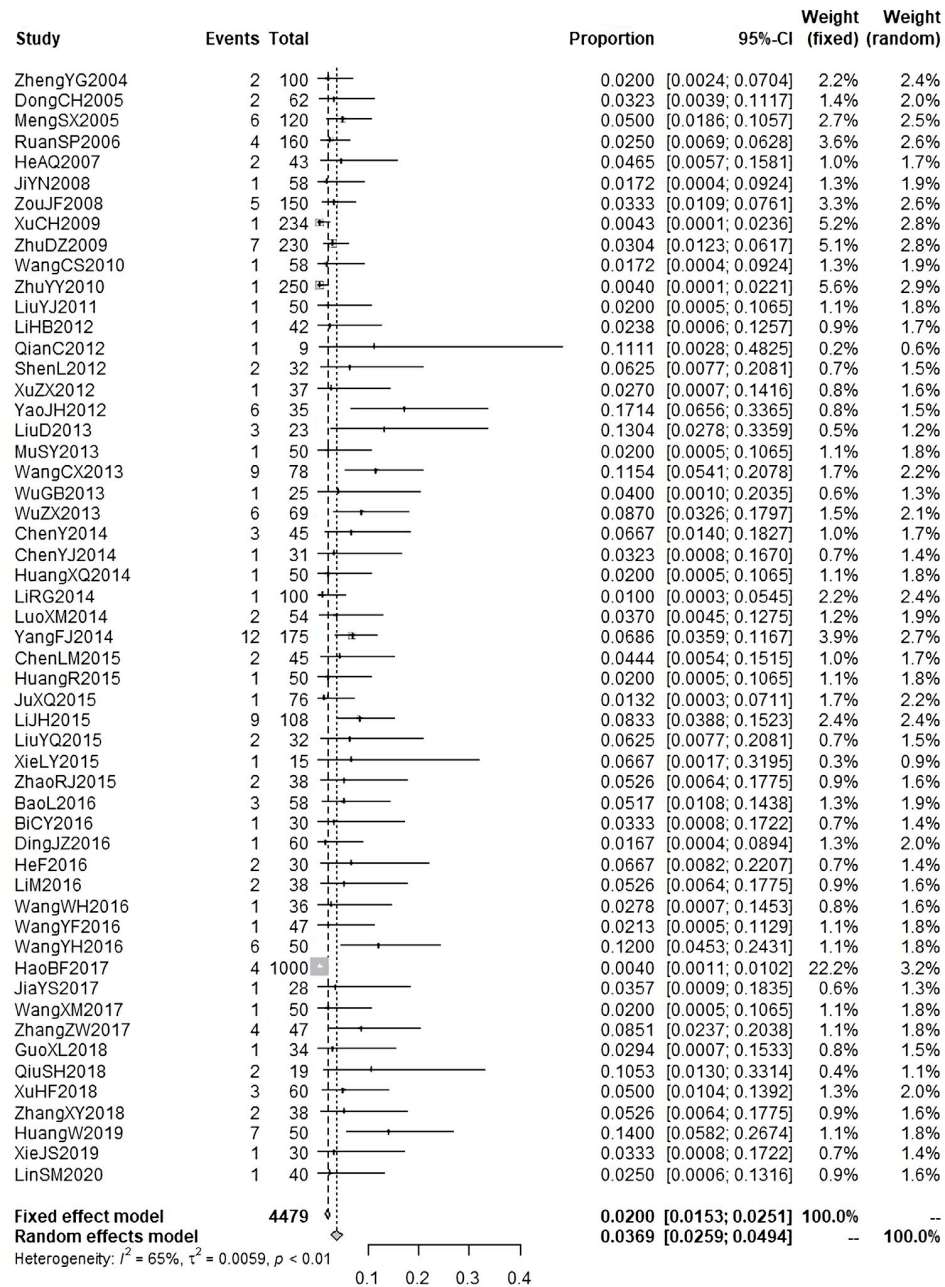
Meta-analysis on ADR incidence of PSDS.

**FIGURE 5:**
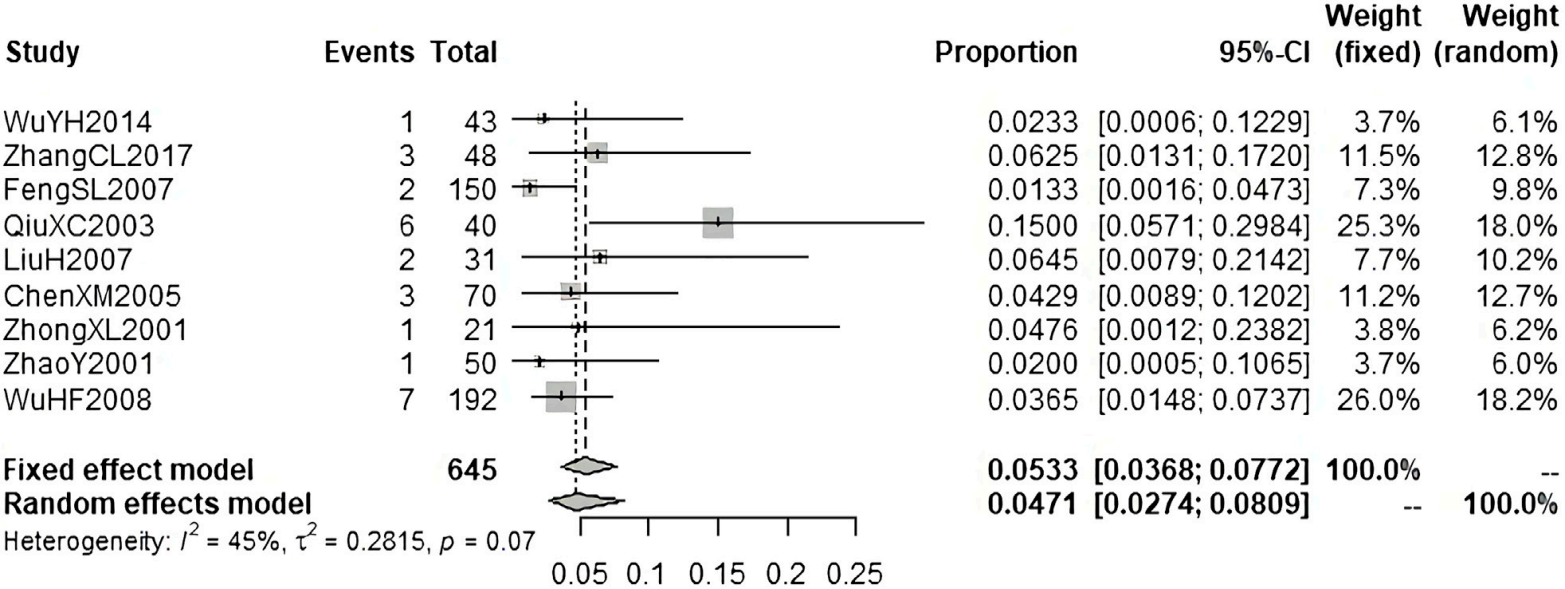
Meta-analysis on ADR incidence of PDS.

According to the results of causality evaluation, the adverse events reported in 23 studies were probably related to the injections, and the adverse events reported in 119 studies were possibly related to the injections. Therefore, all the adverse events reported in these 142 studies were assessed to be ADRs. In these studies, 9490 patients were treated with injections of andrographolide derivatives, of whom 383 patients (4.04%) experienced a total of 383 ADRs. We performed meta-analysis on the ADR incidence of AS, PSDS, and PDS (Figure 3; Figure 4; Figure 5). A total of 3858 patients were treated with AS, of which 198 (5.13%) experienced ADRs. The result of meta-analysis (Figure 3) demonstrated that the overall ADR incidence of AS was 5.48% (95% CI [4.47%, 6.72%], I^2^ = 53%, *p* < 0.01). A total of 4479 patients were treated with PSDS, of which 146 (3.26%) developed ADRs. Meta-analysis (Figure 4) showed that the overall ADR incidence of PSDS was 3.69% (95% CI [2.59%, 4.94%], I^2^ = 65%, *p* < 0.01). A total of 645 patients were treated with PDS, and 26 (4.03%) of them developed ADRs. Meta-analysis (Figure 5) showed that the overall ADR incidence of PDS was 5.33% [95% CI (3.68%, 7.72%), I^2^ = 45%, *p* = 0.07]. For other injections, there was only one corresponding included study respectively (Liang, 1998; Bi, 2006; Ma et al., 2009; Yu et al., 2010), so no meta-analysis on the ADR incidence was performed.

Among the ADRs of andrographolide derivative injections reported in the included RCTs, non-RCTs and case series, 43.34% were gastrointestinal disorders, and 32.38% were skin and subcutaneous tissue disorders. Other ADRs were anaphylaxis, general disorders and abnormal administration site conditions, nervous system disorders, blood and lymphatic system disorders, abnormal medical examination results, and unspecified anaphylactic reaction. According to the information provided in the articles, the severity of 250 (65.27%) ADRs could not be clearly graded, because only the name and number of ADRs were reported, and no other information was provided. For the remaining 133 ADRs, 107 (27.94%) were graded as CTCAE grade 1, 25 (6.53%) were graded as CTCAE grade 2, and one (0.26%) was graded as CTCAE grade 3. Table 1 lists andrographolide derivative injections and their ADR incidence. Table 2; Table 3 shows the details of ADRs of andrographolide derivative injections in the included RCTs, non-RCTs and case series.

**TABLE 1:** ADR incidence of andrographolide derivative injections.

Andrographolide derivative injections	Number of ADRs with CTCAE grading						Total number of ADRs	ADR incidence	Dosage (whether according to instructions)			
	G1	G2	G3	G4	G5	Unclear			Lower	Yes	Higher	Unclear
Andrographolide sulfonate (AS, trade name: Xiyanping injection)	60	9	1	0	0	128	198	5.48% [4.47%, 6.72%]	12	174	6	6
Potassium sodium dehydroandrographolide succinate (PSDS, trade name: Yanhuning injection)	34	16	0	0	0	96	146	3.69% [2.59%, 4.94%]	0	15	6	125
Potassium dehydroandrogrpholide succinate (PDS, trade name: Chuanhuning injection)	9	0	0	0	0	17	26	5.33% [3.68%, 7.72%]	0	13	0	13
Andrographolide sodium bisulfite (ASB, trade name: Lianbizhi injection)	0	0	0	0	0	1	1	0.67% (1/150)	0	0	0	1
*Andrographis paniculata* (AP, trade name: Chuanxinlian injection)	0	0	0	0	0	2	2	3.45% (2/58)	0	0	0	2
Other^a^	0	0	0	0	0	6	6	LiangRJ 1998 Liang, (1998): 15.00% (6/40)	0	0	0	6
	4	0	0	0	0	0	4	YuCC 2010 Yu et al. (2010): 1.54% (4/260)	0	0	0	4

a In these 2 articles, the authors only reported that they used injections containing andrographolide, and did not specify the name of the injection, so the active ingredients of these injections were unknown.

**TABLE 2:** ADRs of andrographolide derivative injections reported in the included RCTs, non-RCTs and case series.

Andrographolide derivative injections	ADRs							
	Gastrointestinal disorders	Skin and subcutaneous tissue disorders	Anaphylaxis	General disorders and abnormal administration site conditions	Nervous system disorders	Blood and lymphatic system disorders	Abnormal investigation (medical examination) results	Unspecified anaphylactic reaction
AS	101	64	11	3	3	1	8	7
PSDS	54	51	0	13	3	0	8	17
PDS	11	6	5	0	4	0	0	0
ASB	0	1	0	0	0	0	0	0
AP	0	2	0	0	0	0	0	0
Other	0	0	4	0	2	0	4	0

**TABLE 3:** Manifestations and severity of ADRs of andrographolide derivative injections reported in the included RCTs, non-RCTs and case series.

ADR manifestations	Number of ADRs with CTCAE grading						Total number of ADRs	Proportion (%)	Detailed description	Dosage (whether according to instructions)			
	G1	G2	G3	G4	G5	Unclear				Lower	Yes	Higher	Unclear
Gastrointestinal disorders (diarrhea, nausea, vomiting, stomachache)	74	11	0	0	0	81	166	43.34	AS: 101	4	89	3	5
									PSDS: 54	0	5	0	49
									PDS: 11	0	5	0	6
Skin and subcutaneous tissue disorders (maculo-papular rash, urticaria, pruritus, flushing)	26	3	0	0	0	95	124	32.38	AS: 64	5	56	3	0
									PSDS: 51	0	0	0	51
									PDS: 6	0	2	0	4
									ASB: 1	0	0	0	1
									AP: 2	0	0	0	2
Anaphylaxis (sweating, chest tightness, palpitation, hypotension, dyspnea, weak pulse, pale complexion)	0	0	1	0	0	19	20	5.22	AS: 11	3	8	0	0
									PDS: 5	0	5	0	0
									Other: 4	0	0	0	4
General disorders and abnormal administration site conditions (pain at injection site, fever, chills, edema, children crying)	0	11	0	0	0	5	16	4.18	AS: 3	0	3	0	0
									PSDS: 13	0	0	0	13
Nervous system disorders (dizziness, headache, seizure, convulsion)	3	0	0	0	0	9	12	3.13	AS: 3	0	3	0	0
									PSDS: 3	0	0	0	3
									PDS: 4	0	1	0	3
									Other: 2	0	0	0	2
Blood and lymphatic system disorders (anemia)	0	0	0	0	0	1	1	0.26	AS: 1	0	1	0	0
Abnormal investigation (Medical examination) results (decreased white blood cell, elevated transaminase)	4	0	0	0	0	16	20	5.22	AS: 8	0	8	0	0
									PSDS: 8	0	0	0	8
									Other: 4	0	0	0	4
Unspecified anaphylactic reaction	0	0	0	0	0	24	24	6.27	AS: 7	0	6	0	1
									PSDS: 17	0	10	6	1
Total	107	25	1	0	0	250	383	100.00					

Among the included RCTs, non-RCTs, and case series using injections of andrographolide derivatives, 11 reported that the ADRs were cured without treatment, 36 reported that the ADRs were cured after stopping the drug and receiving symptomatic treatment, and the remaining 95 studies did not report the prognosis of the ADRs.

In the included case reports, there were 152 patients receiving injections of andrographolide derivatives, of whom 76 (50.00%) got PDS, 41 (26.97%) AS, 28 (18.42%) PSDS, five (3.29%) ASB (Cao, 2006; Wang et al., 2009; Wang et al., 2019), and two (1.32%) other injections (the authors only reported that patients were treated with injection containing andrographolide, but did not specify the name of the injection) (Zhang and Xie, 2008).

In the included case reports, 41 patients were treated with AS, 13 (31.71%) of whom were treated at a dosage according to the instruction. Twenty-five children (60.97%) were treated at the dosage of 50–200 mg, IV, However, the weight of these children was not reported, so it is unclear whether they were treated at an off-label dosage. The dosage of the remaining three patients (7.32%) was not reported. Twenty-eight patients received PSDS, 11 of whom were treated at a dosage according to the instruction. Two adults and one child were treated at off-label doses of 800 and 2000 mg, IV, respectively (De, 2010; Jia, 2013). The dosage of one patient was not reported. For the remaining 13 children, it is unclear whether the injection was used in accordance with the instruction, because there is no specific dosage for children stated in the instruction. Of the 76 patients treated with PDS, 39 (51.32%) were treated according to the instruction. The dosage of nine patients (11.84%) was not reported. For the remaining 28 children (36.84%), it is unclear whether they were treated according to the instruction, because there is no specific dosage for children in the instruction of PDS. Of the five patients receiving ASB, four adults were treated according to the instruction. It is unclear whether one child was treated according to the instruction, since there is no specific dosage for children in the instruction. Among the 152 patients treated with these injections in the included case reports, 133 (87.50%) developed adverse events during the injection or just after the injection, 10 (6.58%) developed adverse events at 2–7 days after the treatment. For the remaining nine patients (5.92%), the time of the adverse events was not reported.

According to the results of causality evaluation, the adverse events of eight patients were certainly related to injections of andrographolide derivatives, 132 were probably related to the injections, 12 were possibly related to the injections. Therefore, adverse events of all these patients were assessed to be ADRs. These 152 patients reported a total of 207 ADRs, of which 97 were anaphylaxis (CTCAE grade 3–5), 41 were skin and subcutaneous tissue disorders (CTCAE grade 1–3), 16 were general disorders and abnormal administration site condition (CTCAE grade 1–3), 15 were gastrointestinal disorders (CTCAE grade 1–3), 13 were abnormal medical examination results (CTCAE grade 2–4). Other ADRs included nervous system disorders (CTCAE grade 1–3), respiratory, thoracic and mediastinal disorders (CTCAE grade 1–3), renal and urinary disorders (CTCAE grade 3), and cardiac disorders (CTCAE grade 2 and 3). Of the anaphylaxis, 55 were life-threatening anaphylactic shock (CTCAE grade 4), and three patients died (CTCAE grade 5) (one each using AS, PSDS and PDS) (Yu, 2003; Jiang and Gong, 2007; Jia, 2013). Two patients developed decreased platelet count (CTCAE grade 4) after using PDS, but the patients were old and had serious heart and respiratory diseases, and eventually died despite emergency rescue efforts (Wang and Zhang, 2003). Among the three patients who were treated at off-label doses of PSDS, two patients (11-year-old girl, 2000 mg, IV; and 21-year-old man, 800 mg, IV) developed severe anaphylaxis (manifested as chest tightness and dyspnea) (De, 2010; Jia, 2013), and the other one patient (60-year-old man, 800 mg, IV) suffered life-threatening anaphylactic shock (manifested as hyperpyrexia, cyanosis, body tremor, and confusion of consciousness) (Jia, 2013). Table 4; Table 5 shows the details of ADRs of andrographolide derivative injections in the included case reports.

**TABLE 4:** ADRs of andrographolide derivative injections reported in the included case reports.

Andrographolide derivative injections	ADRs								
	Anaphylaxis	Skin and subcutaneous tissue disorders	General disorders and abnormal administration site conditions	Gastrointestinal disorders	Nervous system disorders	Respiratory, thoracic and mediastinal disorders	Renal and urinary disorders	Cardiac disorders	Abnormal investigation (medical examination) results
AS	30	12	7	4	1	3	0	0	0
PSDS	21	2	3	7	5	0	0	2	0
PDS	45	27	6	4	2	3	0	3	13
ASB	1	0	0	0	0	0	4	0	0
Other	0	0	0	0	0	0	2	0	0

**TABLE 5:** Manifestations and severity of ADRs of andrographolide derivative injections reported in the included case reports.

ADR manifestations	Number of ADRs with CTCAE grading					Total number of ADRs	Detailed description	Dosage (whether according to instructions)		
	G1	G2	G3	G4	G5			Yes	No	Unclear
Anaphylaxis (anaphylactic reaction, anaphylactic shock)	0	0	39	55	3	97	AS: 30	12	0	18
							PSDS: 21	8	3	10
							PDS: 45	25	0	20
							ASB: 1	0	0	1
Skin and subcutaneous tissue disorders (maculo-papular rash, pruritus, urticaria, purpura)	8	26	7	0	0	41	AS: 12	5	0	7
							PSDS: 2	0	0	2
							PDS: 27	10	0	17
General disorders and abnormal administration site conditions (fever, chills, pain at injection site, edema)	6	9	1	0	0	16	AS: 7	2	0	5
							PSDS: 3	2	0	1
							PDS: 6	1	0	5
Gastrointestinal disorders (nausea, stomachache, vomiting, oral hemorrhage)	5	9	1	0	0	15	AS: 4	0	0	4
							PSDS: 7	3	0	4
							PDS: 4	1	0	3
Nervous system disorders (headache, seizure, convulsion)	1	1	6	0	0	8	AS: 1	0	0	1
							PSDS: 5	1	0	4
							PDS: 2	1	0	1
Respiratory, thoracic and mediastinal disorders (cough, laryngeal edema, epistaxis, wheezing)	1	4	1	0	0	6	AS: 3	0	0	3
							PDS: 3	1	0	2
Renal and urinary disorders (acute kidney injury)	0	0	6	0	0	6	ASB: 4	4	0	0
							Other: 2	0	0	2
Cardiac disorders (cyanosis, myocardial ischemia)	0	2	3	0	0	5	PSDS: 2	1	0	1
							PDS: 3	2	0	1
Abnormal investigation (Medical examination) results (decreased platelet count, decreased white blood cell, elevated transaminase)	0	5	6	2	0	13	PDS: 13	6	0	7

Among these 152 patients, 145 had alleviated ADRs after discontinuation of the drug and symptomatic treatment, and five patients died. Two patients with shock did not recover from shock, and one of them developed multiple organ failure and eventually became vegetative (Li and Zhao, 2014). Three of the dead patients used PDS, one used AS, and one used PSDS. The patient who became vegetative used AS.

In the included studies, two studies (Calabrese et al., 2000; Ciampi et al., 2020) investigated oral andrographolide. In one study (Ciampi et al., 2020), oral andrographolide was used at 140 mg/time, twice daily for patients with not active progressive multiple sclerosis. Adverse events reported in this study include pruriginous rash, dysgeusia, gastroesophageal reflux, which were possibly related to the medication. Other adverse events reported in this study might be caused by diseases of the participants, such as URTI, lower urinary tract infection, gastrointestinal infection, encephalitis, pyelonephritis, acute coronary syndrome, lumbar pain, joint pain, and bursitis. In the other study (Calabrese et al., 2000), the oral andrographolide (PN355) was used in HIV positive patients and normal volunteers at 5 mg/kg, 3 times/day for 3 weeks, escalating to 10 mg/kg, 3 times/day for 3 weeks, and to 20 mg/kg, 3 times/day for a final 3 weeks. Adverse events reported in this study include pruritis/rash, headache, fatigue, loose stools/diarrhea, allergic reaction, bitter taste, tender lymph nodes, nausea, metallic taste, dry tongue, decreased sex drive, eyes sensitive to light, decreased short term memory, dizziness, heartburn, decreased/no taste, and lymphadenopathy.

### Safety of Herbal Preparations of *Andrographis paniculata*

In the included RCTs, non-RCTs, and case series, 10 studies used herbal preparations of AP (Agarwal et al., 2005; Burgos et al., 2009; Saxena et al., 2010; Tang et al., 2011; Sandborn et al., 2013; Phunikhom et al., 2015; Bertoglio et al., 2016; Li and Yang, 2016; Suriyo et al., 2017; Hancke et al., 2019). Two studies were conducted in China, and other studies were conducted in India, Chile, Malaysia, the United States, Canada, Germany, Romania, Ukraine, and Thailand. The herbal preparations used in these 10 studies included six capsules and three tablets. The active ingredient of seven preparations were extract of AP. For the other two preparations, the active ingredient was powder of AP.

According to the results of causality evaluation, the adverse events reported in nine studies were possibly related to the herbal preparations of AP. In one study (Sandborn et al., 2013), some of the reported adverse events were possibly related to the herbal preparation (HMPL-004). Some adverse events might be caused by the disease investigated in the study (for example, abdominal pain and diarrhea might be caused by the investigated disease ulcerative colitis), some might be caused by other diseases of the participants (influenza and nasopharyngitis), and therefore, these adverse events were not possibly related to this herbal preparation. In one study (Suriyo et al., 2017), only the number of adverse events was reported, but the number of patients experiencing adverse events was not reported. Therefore, the incidence in this study could not be obtained. In one study (Hancke et al., 2019), the herbal preparation (active ingredient: *Andrographis paniculata* purified extract) was used in two groups with 300 mg/day and 600 mg/day respectively, and the number of adverse events in the 300 mg/day group was more than that in the 600 mg/day group. In one study (Sandborn et al., 2013), the herbal preparation (active ingredient: *Andrographis paniculata* ethanol extract) was used in two groups with 1200 mg/day and 1800 mg/day respectively, and the adverse events in the two groups were slightly different in manifestation and quantity. In one study (Phunikhom et al., 2015), the herbal preparation (active ingredient: *Andrographis paniculata* extract) was used in two groups at three and five capsules per day, respectively, and the adverse events occurred in both groups were nausea.

The ADRs and AEs of these herbal preparations were mainly gastrointestinal disorders (nausea, diarrhea, vomiting, abdominal pain, dyspepsia, flatulence, constipation) and skin and subcutaneous tissue disorders (rash, pruritis, urticaria). A total of 165 ADRs and AEs of herbal preparations were reported in these studies, of which 43 (26.06%) were graded as CTCAE grade 1, and 4 (2.42%) were graded as CTCAE grade 2. The severity of the remaining 118 (71.52%) ADRs/AEs could not be clearly graded, due to the insufficient information reported in the articles. Since these 10 studies used different preparations, no meta-analysis was performed on the incidence of ADR and AE. The incidence and manifestations of ADRs and AEs were described qualitatively based on what was reported in the original articles. Table 6 shows the detailed information of herbal preparations of AP and ADRs/AEs in the included RCTs, non-RCTs, and case series.

**TABLE 6:** Herbal preparations of *Andrographis paniculata* used in RCTs, non-RCTs, case series and their ADRs/AEs.

Preparations	Form	Active ingredients	Study ID	Country	Dosage	Duration	ADR/AE incidence	Number of ADRs/AEs with CTCAE grading						Total number of ADRs/AEs	Detailed description
								G1	G2	G3	G4	G5	Unclear		
*Andrographis Paniculata* dry powder capsule	Capsule	dry powder of the aerial part of *Andrographis Paniculata*	Agarwal et al. (2005)	Malaysia	start with 600 mg daily, gradually increase to a maximum of 1.8 mg daily	12 weeks	5.00% (1/20)	1	0	0	0	0	0	1	nausea 1
KalmCold	Capsule	extract from the leaves of *Andrographis Paniculata* Nees	Saxena et al. (2010)	India	200 mg/day	5 days	5.36% (6/112)	7	1	0	0	0	0	8	diarrhea 3, vomiting 1, epistaxis 1, urticaria 1, nausea 1, lethargy 1
ApE tablet	Tablet	*Andrographis Paniculata* purified extract	Bertoglio et al. (2016)	Chile	1 tablet/time, twice daily	12 months	7.69% (1/13)	1	0	0	0	0	0	1	skin rash 1
Chuanxinlian tablets	Tablet	*Andrographis Paniculata*	Li and Yang, (2016)	China	2–3 tablets/time, 3–4 times/day	7–14 days	3.50% (7/200)	0	0	0	0	0	7	7	rash 3, digestive symptoms 2, dizziness 1, fever 1
Standardized *Andrographis Paniculata* capsule	Capsule	*Andrographis Paniculata* crude powder	Suriyo et al. (2017) ^a^	Thailand	4 capsules/time, 3 times/day	3 days	—	24	2	0	0	0	4	30	thirst 8, dysgeusia 2, abdominal swelling 3, skin or eyes turn yellow 3, itchiness 1, abdominal pain 2, fever 1, dizziness 1, nausea 1, shiver 1, loss of weight 3, fatigue 4
FANG(30)	Tablet	dried extract of *Andrographis paniculata*	Burgos et al. (2009)	Chile	1 tablet/time, 3 times/day	14 weeks	36.67% (11/30)	0	0	0	0	0	11	11	headache 3, diarrhea 1, nausea 2, stomach discomfort 1, fatigue 1, common cold 1, pruritus/rash 1, cramps 1
ParActin	Capsule	*Andrographis paniculata* purified extract	Hancke et al. (2019)	India	300 mg/day	12 weeks	21.62% (8/37)	0	0	0	0	0	9	9	acidity 4, elevated alanine aminotrasferase 2, constipation 1, oral ulcers 1
					600 mg/day		2.86% (1/35)								elevated alanine aminotrasferase 1
HMPL-004	Capsule	*Andrographis Paniculata* ethanol extract	Tang et al. (2011)	China	1200 mg/day	8 weeks	13.21% (7/53)	0	0	0	0	0	9	9	aphthous ulcer 1, white blood cell decrease 1, abdominal pain 1, blood in stool 1, fever 1, elevated glucose 1, rash 1, blood in urine 1, elevated C-reactive protein 1
			Sandborn et al. (2013)	the United States, Canada, Germany, Romania, Ukraine	1200 mg/day	8 weeks	60.00% (45/75)	5	1	0	0	0	78	84	headache 8, abdominal pain 4, nausea 4, diarrhea 3, dyspepsia 3, ageusia 3, alanine aminotransferase increased 3, blood alkaline phosphatase increased 3, gamma-glutamyl transferase increased 3, rash 3, influenza 2, nasopharyngitis 2, fatigue 2, flatulence 1
					1800 mg/day		52.00% (39/75)								abdominal pain 4, diarrhea 4, flatulence 4, headache 4, nausea 3, dysgeusia 3, blood glucose increased 3, rash 3, back pain 3, ageusia 2, influenza 2, nasopharyngitis 2, dyspepsia 1, gamma-glutamyl transferase increased 1, anemia 1
*Andrographis paniculata* extract (APE) capsule	Capsule	*Andrographis paniculata* extract	Phunikhom et al. (2015)	Thailand	3 capsules/day	8 weeks	10.00% (2/20)	5	0	0	0	0	0	5	nausea 2
					5 capsules/day		15.00% (3/20)								nausea 3

a In this study, only the number of AEs was reported, but the number of patients experiencing AEs was not reported. Therefore, the incidence of AEs could not be obtained.

Among these 10 studies, one study reported that the ADRs were cured without treatment (Saxena et al., 2010), and two studies reported that the ADRs were cured after discontinuing the drug and receiving symptomatic treatment (Agarwal et al., 2005; Bertoglio et al., 2016). The remaining seven studies did not report the prognosis.

In the included case reports, three patients took herbal preparation Chuanxinlian tablets at a dosage of 5 tablets/time, 3 times/day (Fan, 1992; Liu and Hu, 2003). One of them experienced adverse events after taking the medicine twice, and the other two patients experienced adverse events at 30 min after taking the medicine. According to the results of causality evaluation, the adverse events of all these three patients were probably related to the preparation, and therefore were judged to be ADRs. The ADRs of one patient manifested as rash and skin itching (CTCAE grade 3), and the other two manifested as dizziness (CTCAE grade 1). The ADRs of these three patients were alleviated after drug discontinuance and symptomatic treatment.

## Discussion

### Summary of Findings

This systematic review included 262 clinical studies that reported ADRs and AEs of andrographolide derivative medications and herbal preparations of AP. The included studies mainly investigated three injections of andrographolide derivatives: AS, PSDS and PDS. The results of meta-analysis showed that the ADR incidence of AS, PSDS, and PDS were 5.48, 3.69 and 5.33%, respectively. The ADRs of andrographolide derivative injections are mainly gastrointestinal disorders, skin and subcutaneous tissue disorders, followed by anaphylaxis, general disorders and abnormal administration site conditions, nervous system disorders. The majority of these ADRs are mild to moderate, and a small number of patients may experience severe or life-threatening ADRs. Most of these ADRs caused by andrographolide derivative injections can be alleviated after discontinuation of the drug and symptomatic treatment. In the included case reports, one patient developed anaphylactic shock after using AS, and eventually turned into vegetative state. Five patients died after using injections of andrographolide derivatives (three used PDS, one each used AS and PSDS). The included studies involved 9 herbal preparations of AP (tablets and capsules). The ADRs and AEs of these herbal preparations are mainly mild to moderate gastrointestinal disorders, skin and subcutaneous tissue disorders.

### Comparison With Previous Studies

There have been other clinical studies and literature studies on the safety of other drugs used to treat RTIs. A meta-analysis evaluating the effect of clarithromycin on streptococcal pharyngitis included five studies, in which 600 patients were treated with clarithromycin (Hoban and Nauta, 2019). Among these patients, 216 (36%) patients had AEs, of which 51 (8.50%) had AEs that were probably related to the use of clarithromycin. Echinacea preparations are commonly used to prevent and treat URTI. A systematic review evaluating the effect of Echinacea preparations on preventing and treating URTI included 16 studies, in which 1644 patients used Echinacea preparations, and 279 (16.97%) had AEs (David and Cunningham, 2019). Kan Jang oral solution is an herbal medicinal product for URTI. In an RCT using Kan Jang oral solution to treat acute URTI, 66 patients used Kan Jang oral solution, of which two (3.03%) patients had AEs (Barth et al., 2015). In an RCT investigating the antipyretic effect of ibuprofen in children with URTI, 85 children were treated with ibuprofen, of which 10 (11.76%) had ADRs (Yoon et al., 2008). Qingkailing injection is a traditional Chinese medicine injection that can be used to treat URTI and pneumonia. A meta-analysis of the ADR incidence of Qingkailing injection showed that the incidence of ADRs in skin and mucosa system was 2%, in digestive system was 6%, and at injection site was 4% (Ai et al., 2015). Meta-analysis in this study showed that the ADR incidence of AS, PSDS and PDS was 5.48, 3.69 and 5.33%, respectively. Compared with other drugs for URTI and other antipyretic drugs, these injections of andrographolide derivatives have low ADR incidence. Nevertheless, three patients in the included studies developed lethal anaphylactic shock (CTCAE grade 5) after treated with injections of andrographolide derivatives (AS, PSDS and PDS). These injections should be used with caution.

There is currently no comprehensive systematic review on the safety of andrographolide derivative medications and herbal preparations of AP. Some published studies have reviewed the ADRs of several andrographolide derivatives. Two studies reviewed the ADRs of four andrographolide derivative injections (AS, PSDS, PDS, ASB) (Wang et al., 2011; Xiang et al., 2016). According to the results of these two studies, the ADRs of these andrographolide derivative injections were mainly skin and subcutaneous tissue disorders, systemic manifestations (fever, chills, anaphylactic reaction and anaphylactic shock), digestive disorders. These two studies reviewed the ADRs of four andrographolide derivative injections, but did not clearly indicate whether they distinguished the included studies according to study type. In this study, we found that different types of studies have different reports on ADRs. For example, in RCTs, non-RCTs and case series, the authors usually report a small number of ADRs occurred in all patients who took the drug, and these data can be used to calculate the incidence of ADRs. In case reports, the authors report the ADRs occurred in one patient who took the drug, and these ADRs are usually more serious. Therefore, in this study, we separated these two parts of studies, and reported the occurrence of ADRs respectively. The results of this study showed that the ADRs of andrographolide derivative injections were mainly gastrointestinal disorders, skin and subcutaneous tissue disorders, followed by anaphylaxis, general disorders and abnormal administration site conditions. The ADRs reported in case reports were mainly anaphylactic reactions and anaphylactic shock. Moreover, in this study, we performed single-rate meta-analysis for the ADR incidence of AS, PSDS and PDS. For herbal preparations of AP, no previous studies have reviewed the safety of these preparations, and this study fills the gap.

### Implications for Clinical Practice and Future Research

In the included case reports, 97 patients developed anaphylaxis after being treated with injections of andrographolide derivatives, of which 55 patients had anaphylaxis that were life-threatening (CTCAE grade 4), and three patients died (CTCAE grade 5). Before prescribing medicine, doctors should inquire the allergic history of patients carefully, and patients who are allergic to the preparation should carefully take the medicine to avoid serious ADRs and AEs. Some of the ADRs caused by andrographolide derivative injections are severe, so they should be used with caution, and possible ADRs should be closely monitored.

Treatment dosage is of importance to ADRs. Five injections of andrographolide derivatives were involved in this study, of which only AS specifies the dosage for children in the instruction. Many of the included clinical studies were for children. It is unclear whether the other andrographolide derivative injections were used at an off-label dosage for children in the included studies, because dosage for children is not specified in the instructions. In clinical practice, these preparations may be more likely to overdose when applied to children, because there is no prescribed dosage for children in the instructions. Therefore, if dosages for children are supplemented in the instructions, the safe application of these preparations in children will be promoted.

Except for a few studies, the included studies have methodological deficiencies. Moreover, except for case reports, other studies usually report the information on ADRs and AEs very briefly, which will make it more difficult for readers to clearly assess the severity of ADRs and AEs, and to evaluate the causal relationship. More studies with higher methodological quality are needed to provide more high-quality evidence for evaluating the safety of andrographolide derivatives, while herbal preparations of AP can generally be regarded as safe. The CONSORT for Harms checklist is a checklist applicable for reporting information related to harms in RCTs (Ioannidis et al., 2004), which includes nine recommended items, involving title and abstract, introduction, methods, results, and discussion. In order to report more standardized and adequate information about ADRs and AEs, we recommend that authors refer to this checklist for reporting.

### Strengths and Limitations

Clinical studies that reported the ADRs and AEs of andrographolide derivative medications and herbal preparations of AP were comprehensively searched and included in this study. Moreover, the severity of ADRs and AEs were graded using the CTCAE system. The number, manifestations and severity grading of ADRs and AEs were all reported in this study. Therefore, this systematic review provides comprehensive information on the safety of andrographolide derivative medications and herbal preparations of AP.

The limitations of this study are, first of all, most of the included studies have methodological deficiencies, and the overall quality of this study is limited by the quality of original studies. Secondly, we only included studies that reported the occurrence of ADRs and AEs, but did not include studies that clearly reported that patients did not have ADRs or AEs after taking andrographolide derivative medications and herbal preparations of AP. Hence, the ADR incidence of andrographolide derivative injections resulted from meta-analysis may be slightly higher than the actual ADR incidence. Thirdly, due to the large variety and wide application of andrographolide derivative medications and herbal preparations of AP, the number of clinical studies using these preparations is very large. Studies using other drugs in addition to andrographolide derivative medications, herbal preparations of AP, and symptomatic treatment were excluded from this study. In this way, the influence of other drugs on the results of causality evaluation could be avoided, but at the same time, the included studies would not be comprehensive enough. Furthermore, this study only summarized the information on ADRs and AEs obtained from literature. There is a lack of information from ADR monitoring centers (such as WHO-UMC, and National Center for ADR Monitoring, China). As a result, information on the ADRs of these preparations reported in this study is not comprehensive enough. Moreover, the severity of ADRs and AEs were graded only based on the information reported in the articles. However, except for case reports, other studies only briefly reported the information about ADRs and AEs, and therefore the severity of many ADRs and AEs could not be clearly graded. And that, the severity grading was solely based on the information provided in the articles, so the results should be interpreted and applied with caution.

## Conclusion

The ADR incidence of three most used injections of andrographolide derivatives (AS, PSDS, PDS) are 5.48, 3.69 and 5.33%, respectively. The ADRs of andrographolide derivative injections are mainly gastrointestinal disorders, skin and subcutaneous tissue disorders, followed by anaphylaxis, general disorders and abnormal administration site conditions, etc. Most of the ADRs are mild, moderate, or medically significant (CTCAE grade 1–3). Based on data from this review, we recommend that AS, PSDS and PDS be used with caution, because a small number of patients can experience life-threatening or lethal anaphylactic shock (CTCAE grade 4 and 5) after using these injections. This is of particular importance in patients with a history of allergy. Possible ADRs should be closely monitored. Herbal preparations of AP are essentially safe. Most of the included clinical studies have limited methodological quality. More post-marketing studies on the safety of andrographolide derivative medications are needed.

## Data Availability

The original contributions presented in the study are included in the article/Supplementary Material, further inquiries can be directed to the corresponding author.
