# Supplementary material for: Adverse Effects of Andrographolide Derivative Medications Compared to the Safe use of Herbal Preparations of Andrographis paniculata: Results of a Systematic Review and Meta-Analysis of Clinical Studies
Source: Front Pharmacol. 2022 Jan 28;13:773282. doi: 10.3389/fphar.2022.773282 (PMC8831758; doi:10.3389/fphar.2022.773282)
Supplement: Supplementary file 1 [file Table1.DOCX]

**Supplementary material**

**Search strategy of Pubmed**

#1 MeSH Terms: andrographis

#2 Title/Abstract: (andrographis) OR (andrographolide) OR (chuanxinlian)

#3 #1 OR #2

#4 MeSH Terms: drug-related side effects and adverse reactions

#5 Text word: (adverse drug reaction) OR (adverse event) OR (adverse effect) OR (adverse reaction) OR (side effect) OR (complication) OR (safety) OR (toxicity)

#6 #4 OR #5

#7 #3 AND #6
